# Variation in Antiviral Protection Mediated by Different *Wolbachia* Strains in *Drosophila simulans*

**DOI:** 10.1371/journal.ppat.1000656

**Published:** 2009-11-13

**Authors:** Sheree E. Osborne, Yi San Leong, Scott L. O'Neill, Karyn N. Johnson

**Affiliations:** School of Biological Sciences, The University of Queensland, St Lucia, Queensland, Australia; Stanford University, United States of America

## Abstract

Drosophila C virus (DCV) is a natural pathogen of *Drosophila* and a useful model for studying antiviral defences. The *Drosophila* host is also commonly infected with the widespread endosymbiotic bacteria *Wolbachia pipientis*. When DCV coinfects *Wolbachia*-infected *D. melanogaster*, virus particles accumulate more slowly and virus induced mortality is substantially delayed. Considering that *Wolbachia* is estimated to infect up to two-thirds of all insect species, the observed protective effects of *Wolbachia* may extend to a range of both beneficial and pest insects, including insects that vector important viral diseases of humans, animals and plants. Currently, *Wolbachia*-mediated antiviral protection has only been described from a limited number of very closely related strains that infect *D. melanogaster*. We used *D. simulans* and its naturally occurring *Wolbachia* infections to test the generality of the *Wolbachia*-mediated antiviral protection. We generated paired *D. simulans* lines either uninfected or infected with five different *Wolbachia* strains. Each paired fly line was challenged with DCV and Flock House virus. Significant antiviral protection was seen for some but not all of the *Wolbachia* strain-fly line combinations tested. In some cases, protection from virus-induced mortality was associated with a delay in virus accumulation, but some *Wolbachia*-infected flies were tolerant to high titres of DCV. The *Wolbachia* strains that did protect occurred at comparatively high density within the flies and were most closely related to the *D. melanogaster Wolbachia* strain *w*Mel. These results indicate that *Wolbachia*-mediated antiviral protection is not ubiquitous, a finding that is important for understanding the distribution of *Wolbachia* and virus in natural insect populations.

## Author Summary

Many human, animal and plant viruses are transmitted between hosts by insect vectors. Understanding the processes that control virus infection in insects may facilitate strategies that aim to control the spread of important viral pathogens. Infection of the model insect *Drosophila* with the endosymbiotic bacteria *Wolbachia* can significantly affect the outcome of infection with pathogenic viruses. *Wolbachia* is widespread in insects, so here we tested the generality of antiviral protection across diverse strains of the bacteria. We show that some, but not all, strains of *Wolbachia* protect flies from pathogenic viruses. These results have implications for proposed strategies utilising *Wolbachia* to control the spread of insect-transmitted viral diseases, such as dengue.

## Introduction

As obligate intracellular parasites, viruses have intricate associations with their hosts. Many viruses have deleterious effects on their host including virus induced pathology, morbidity and mortality. For this reason a suite of antiviral defence responses have evolved. Some of these responses are conserved across different kingdoms, while others are unique to closely related groups of organisms. For example, viruses that infect insects encounter some host defences that are distinctive to invertebrates, such as the peritrophic matrix.

There are a number of motivations for studying antiviral responses in insects. Insects are a useful model for research on innate immune responses, and because of the evolutionary conservation in many of these pathways, this research may lead to an increased understanding of antiviral immunity in mammals (reviewed in [1]). It is also important to understand insect antiviral responses for other reasons. Viruses cause diseases in both pest insect species and beneficial insects. Also insects are involved in the transmission of many viruses that cause serious disease in humans, other animals and plants. Thus there are diverse reasons for wanting to control virus infection in insects and understanding antiviral responses in insects may facilitate strategies to achieve this.

The vinegar fly, *Drosophila melanogaster*, is an appropriate model for the study of antiviral responses. The *Drosophila* cellular antiviral responses include both the intrinsic RNAi pathway and inducible immune pathways [2]–[6]. In addition to host antiviral defences, *D. melanogaster* are also protected from RNA viruses when infected by the intracellular bacterium, *Wolbachia pipientis* [7],[8]. In *D. melanogaster* the interaction between *Wolbachia* and virus has important implications for the outcome of viral infection.

Recent studies on antiviral responses in *Drosophila* have utilised the most pathogenic of the *Drosophila* viruses, Drosophila C virus (DCV). A member of the *Dicistroviridae* family, DCV is a natural pathogen of *D. melanogaster* found in both wild and laboratory fly populations [9],[10]. Following injection of DCV into the hemocoel of adult *D. melanogaster,* flies typically die within 4–6 days [11]. In contrast, following injection of DCV into *Wolbachia* infected flies, the accumulation of infectious DCV particles is delayed and flies live for 12–14 days [7],[8]. *Wolbachia*-mediated antiviral protection is not limited to DCV. *Wolbachia* infection also protects flies from mortality induced by a second member of the *Dicistroviridae* family *Cricket paralysis virus* (CrPV) and a member of the *Nodaviridae* family *Flock House virus* (FHV) [7],[8]. In addition, antiviral protection has been demonstrated in a number of *D. melanogaster* genetic backgrounds and using closely related *Wolbachia* strains that naturally occur in *D. melanogaster,* namely *w*MelCS and *w*MelPop [7],[8].

*Wolbachia* are predicted to infect from 20–70% of insect species [12]–[14], which raises the possibility that *Wolbachia* may potentially influence virus infection across a large number of insect species. Bacteria of the genus *Wolbachia* are maternally inherited intracellular symbionts, which are best known for their propensity to manipulate host reproductive systems [15]. *Wolbachia* infect a wide range of arthropods and filarial nematodes and are classified into 7–8 phylogenetic supergroups based on analysis of the sequence of a number of *Wolbachia* genes (see [16] and references therein). The majority of known *Wolbachia* strains that infect insect species belong to either supergroup A or B [17],[18]. The *Wolbachia* that occur in *D. melanogaster* are very closely related strains from the Mel clade of supergroup A [19].

It is currently not known whether antiviral protection is mediated by diverse strains of *Wolbachia*. The fly species, *D. simulans* is infected by up to six strains of *Wolbachia* that span across both supergroup A and B [18],[20], including three supergroup A strains *w*Au, *w*Ri and *w*Ha and one supergroup B strain *w*No [18],[20]. Here we tested whether *Wolbachia*-mediated protection extends to insects other than *D. melanogaster* and whether each of the *Wolbachia* strains could protect *D. simulans* from virus infection. Our results show that some, but not all, of the *Wolbachia* strains protected naturally infected *D. simulans* lines from virus-induced mortality.

## Results

### *Wolbachia* strain *w*Mel can protect *D. simulans* from DCV

**Figure 1:**
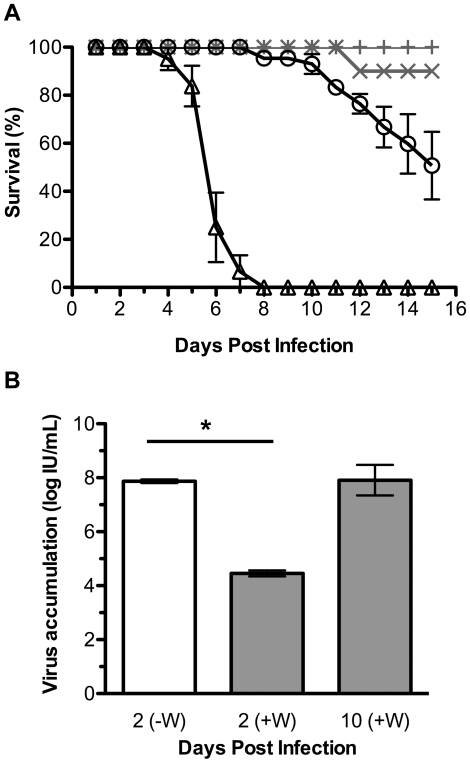
*Wolbachia* strain *w*Mel provides antiviral protection in *D. simulans*. (**A**) Graph shows survival of flies infected with DCV (black line) or mock infected (grey line). *w*Mel-infected (circle and plus sign) or uninfected (triangle and cross) flies. The survival of DCV infected flies with and without *Wolbachia* is significantly different (p<0.0001). Error bars represent SEM calculated from three replicate vials. This is a representative experiment which was repeated twice more with similar results. (**B**) Graph showing accumulation of infectious DCV in *w*Mel infected (grey bars) or uninfected (white bar) flies. Bars represent means from two replicates with SEM shown, and * indicates a significant difference between the means of day 2 samples (p<0.05, unpaired t test).

*Wolbachia* strains closely related to *w*Mel have previously been shown to protect their natural host *D. melanogaster* from accumulation of DCV particles and DCV-induced mortality [7],[8]. To establish whether *w*Mel can protect *D. simulans* from DCV, we assayed Me29, a *D. simulans* line that was transinfected with *w*Mel [21] (Table 1). Me29 flies infected with *w*Mel and the genetically paired population that had been cured of *Wolbachia* infection were challenged with DCV and mortality was recorded for 15 days (Figure 1A). For flies both with and without *Wolbachia* the mortality in PBS injected controls was negligible. All DCV injected *w*Mel-free flies died by 8 days post infection (dpi), with a median survival time of 6 days. In contrast, at 15 dpi about 50% of *w*Mel infected flies remained alive. These results indicate that the presence of *w*Mel mediates a significant decrease in DCV induced mortality in Me29 flies.

**Table 1:** Fly lines and *Wolbachia* strains.

*Drosophila simulans* line	*Wolbachia* strain	Reference
Me29	*w*Mel	Poinsot et al., 1998 [21]
CO	*w*Au	Hoffmann et al., 1996 [54]
DSR	*w*Ri	Hoffmann et al., 1986 [45]
DSH	*w*Ha	O'Neill & Karr, 1990 [47]
N7NO	*w*No	Mercot & Poinsot, 1998 [46]

The accumulation of infectious DCV particles was assayed in Me29 flies with and without *w*Mel. The titre of infectious virus in homogenates from flies collected 2 dpi was significantly different in flies with and without *w*Mel (p<0.002; Figure 1B). The titre of virus in flies without *Wolbachia* was estimated to be about 2600-fold greater than in Me29 flies infected with *w*Mel. By 10 dpi there were no surviving *Wolbachia*-free flies and the virus titre in the surviving *w*Mel infected flies had increased to a level similar to that of *Wolbachia*-free flies at 2 dpi. This indicates that the presence of *w*Mel in Me29 flies delays rather than prevents DCV accumulation.

### *D. simulans Wolbachia* strains and protection from DCV induced mortality

**Figure 2:**
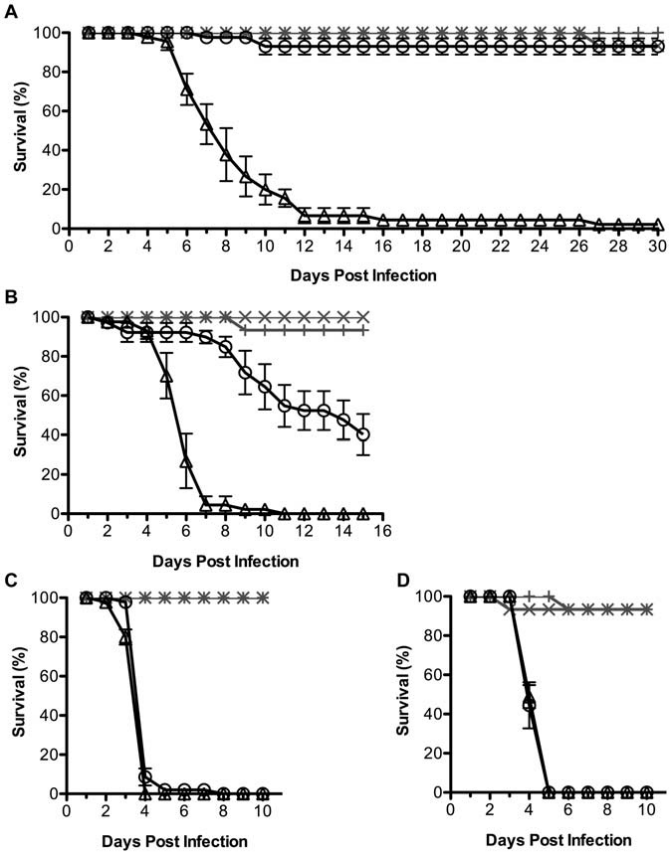
Antiviral protection of different *Wolbachia* strains in *D. simulans*. Graphs show survival of flies infected by *w*Au (**A**), *w*Ri (**B**), *w*Ha (**C**), and *w*No (**D**) challenged with DCV (black line) or mock infected (grey line). Flies with *Wolbachia* (circle and plus sign) and without *Wolbachia* (triangle and cross). Error bars represent SEM calculated from three replicates. The survival of DCV infected flies with and without *Wolbachia* is significantly different for *w*Au (p<0.0001), *w*Ri (p<0.0001), and *w*Ha (p<0.01), using log rank test on Kaplan-Meier curves. Experiments were replicated on at least two additional independent cohorts of flies, and the results for all respective replicates of experiments shown in panel A, B and D were similar, however the replicates for panel C varied (see Results).

*D. simulans* populations are naturally infected with a range of *Wolbachia* strains. To analyse whether diverse strains could protect from DCV induced mortality we assayed four *D. simulans* lines CO, DSR, DSH and N7NO, which are naturally infected with *w*Au, *w*Ri, *w*Ha and *w*No, respectively (Table 1). Each of the four fly lines was treated with tetracycline to produce a genetically paired line without *Wolbachia* infection. Flies with and without *Wolbachia* were challenged by injection with DCV or mock infected with PBS (Figure 2). In all cases less than 10% mortality occurred in the mock-infected flies, indicating that in the absence of virus fly survival was stable over the course of the experiments. The CO flies without *Wolbachia* had a median survival time of 8 days following DCV injection (Figure 2A). Strikingly, the *w*Au-infected CO flies survived DCV infection; more than 90% were alive when the experiment was terminated at 30 dpi. The *w*Ri-infected DSR flies had significantly better survival (p<0.0001) than *Wolbachia*-free DSR flies (Figure 2B). The median survival times following DCV infection were 14 dpi as compared to 6 dpi for flies with and without *w*Ri, respectively. Thus presence of either *w*Au or *w*Ri in *D. simulans* can mitigate DCV-induced mortality.

Not all *Wolbachia* strains protected flies from DCV induced mortality. The median survival time of DSH and N7NO flies challenged with DCV was 4 days regardless of *Wolbachia* infection status for fly lines infected by *w*Ha or *w*No, respectively (Figure 2C and 2D). While there was a small but statistically significant (p = 0.001) difference between the survival curves for the DSH flies with and without *w*Ha infection for the representative experiment shown in Figure 2C, a significant difference was evident in only 2 out of 4 experiments replicated on independent cohorts of flies (data not shown). Taken together, the minor difference in survival and non-reproducible nature of the result suggests that it is unlikely that this difference is biologically relevant, and as such we interpret the results as indicating that there is no protection against DCV induced mortality in the DSH flies infected with *w*Ha. There was no difference between the survival curves of N7NO flies with and without *w*No infection (p = 0.7). To investigate whether protection would be evident for these lines challenged with reduced amounts of virus we decreased the concentration of DCV injected by 10- or 100-fold. Even at these lower doses of virus no *Wolbachia*-mediated antiviral protection was observed in DSH and N7NO flies (data not shown).

### Accumulation of DCV in flies with and without *Wolbachia*

**Figure 3:**
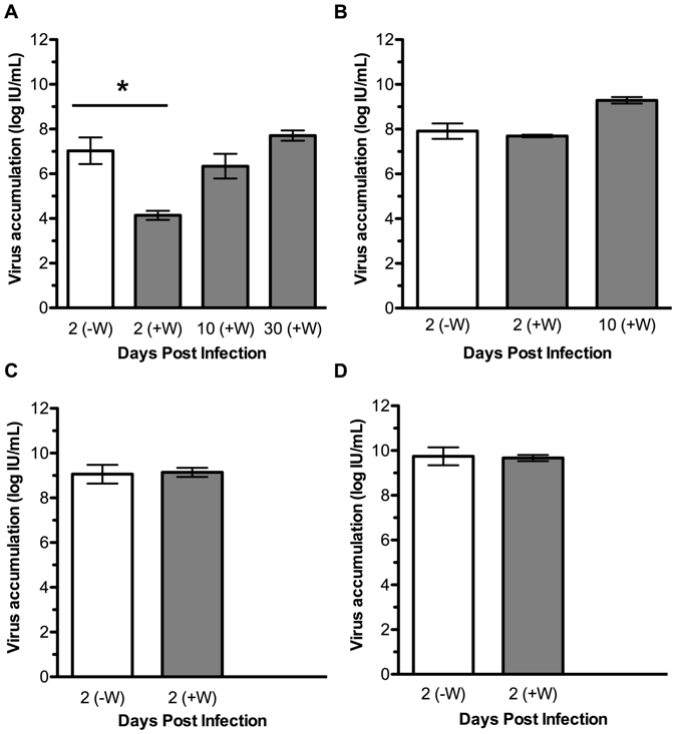
The effect of different *Wolbachia* strains on the accumulation of DCV in *D. simulans*. Graphs show accumulation of infectious DCV in flies with (grey bar) or without (white bar) *w*Au (**A**), *w*Ri (**B**), *w*Ha (**C**), and *w*No (**D**). Bars represent means from two replicates with SEM shown, and * indicates a significant difference between the means of day 2 samples (p<0.05, unpaired t test).

DCV accumulation was assayed in each *D. simulans* line in the presence or absence of *Wolbachia* (Figure 3). DCV infected flies were assayed at 2 dpi and the DCV titre was compared for each fly line with and without *Wolbachia* infection. The average DCV titre was approximately 800-fold lower in CO flies infected with *w*Au compared to paired *Wolbachia*-free flies, and an unpaired t test showed this to be a significant difference (p<0.05; Figure 3A). Interestingly, although *w*Au infected flies survived DCV infection (Figure 2A), virus continued to accumulate beyond 2 dpi and high titres of DCV were observed in *w*Au-infected flies harvested at both 10 and 30 dpi (Figure 3A). This shows that these flies did not clear the virus infection. The titre of DCV was similar when comparing flies with and without *Wolbachia* at 2 dpi for each of the three other fly lines assayed (Figure 3B–D).

### *D. simulans Wolbachia* strains and protection from FHV induced mortality

**Figure 4:**
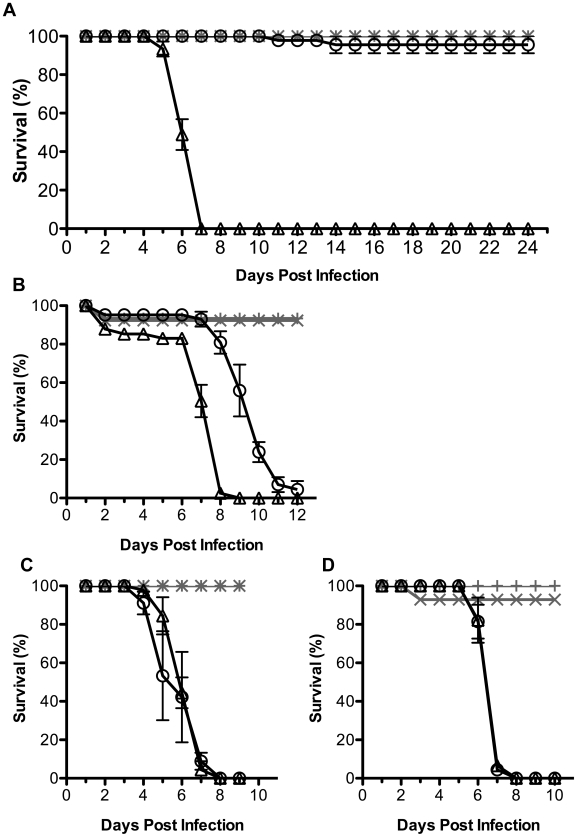
The effect of different *Wolbachia* strains on the accumulation of FHV in *D. simulans.* Graphs show survival of flies infected by *w*Au (**A**), *w*Ri (**B**), *w*Ha (**C**), and *w*No (**D**) challenged with FHV (black line) or mock infected (grey line). *Wolbachia* infected (circle and plus sign) and uninfected (triangle and cross) flies. Error bars represent SEM calculated from three replicates. The survival of FHV infected flies with and without *Wolbachia* is significantly different for *w*Au and *w*Ri (p<0.0001, log rank test on Kaplan-Meier curves). For each fly line a similar result was recorded in a replicate experiment.

Having identified that some but not all *Wolbachia* strains mediate protection against DCV in the *D. simulans* lines tested, we next investigated whether antiviral protection was consistent across different viruses. Flies with and without *Wolbachia* were challenged by injection with FHV or mock infected with PBS (Figure 4). In all cases mortality in the mock-infected control flies was negligible. The CO flies without *Wolbachia* infection reached 100% mortality within 7 days of injection with FHV (Figure 4A). Similar to challenge with DCV the *w*Au-infected flies survived FHV infection; more than 90% were alive when the experiment was terminated at 24 dpi. The *w*Ri-infected DSR flies had significantly better survival (p<0.0001) than *Wolbachia*-free DSR flies (Figure 4B). The median survival times or DSR flies challenged with FHV were 10 days as compared to 7 days with and without *w*Ri, respectively. Thus median time to death was reduced in both DCV and FHV infections for *w*Ri-infected DSR flies. No virus-induced mortality was observed in *w*Au-infected CO flies for either virus.

Not all of the fly lines were protected from FHV-induced mortality by *Wolbachia* infection. The median survival time of DSH flies challenged with FHV was 6 days regardless of the presence or absence of *w*Ha (Figure 4C) and there was no significant difference in the survival curves (p = 0.4). For the N7NO line there was no difference between the survival curves with and without *w*No infection (p = 0.5; Figure 4D).

### *Wolbachia* density in fly lines

**Figure 5:**
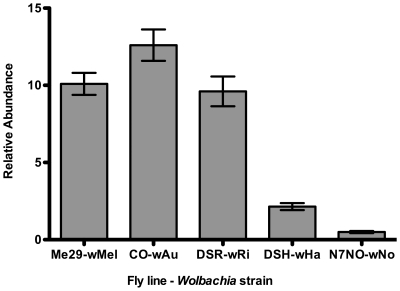
Relative-density of *Wolbachia* strains in *D. simulans*. For each fly line the graph shows the relative abundance of *Wolbachia* to host genomic DNA estimated using quantitative PCR. Bars represent the mean of 10 replicates and error bars are SEM.

To investigate whether virus protection correlated with the density of the *Wolbachia* in the fly lines, we utilized quantitative PCR to determine *Wolbachia* density from pools of 5 male flies from each fly line. Estimates of abundance for a single copy *Wolbachia* gene were determined and then normalized against abundance of a single copy host gene to determine relative abundance of *Wolbachia* (Figure 5). The three *Wolbachia* strains (*w*Mel, *w*Ri and *w*Au ) that gave strong antiviral protection in the *D. simulans* lines, were significantly more abundant in these flies than the strains that gave no protection (*w*Ha and *w*No).

## Discussion

Many insect species are infected with *Wolbachia*, raising the possibility that *Wolbachia*-mediated antiviral protection could be a widespread phenomenon. *Wolbachia* strains vary both between host species and within a host species (for example [20]). Naturally occurring *Wolbachia* strains in *D. melanogaster* ubiquitously protect against DCV [7],[8], however these strains are very closely related [19]. *Wolbachia* is maternally inherited and therefore has a close association with its host. Using *D. simulans* fly lines that are naturally infected by different *Wolbachia* strains we showed that some strains did not mitigate virus-induced mortality. Strains *w*Au and *w*Ri protected the CO and DSH host flies respectively. In contrast, neither *w*Ha nor *w*No protected their host lines from DCV induced mortality. Phylogenetic analysis indicates that the *D. simulans Wolbachia* strains *w*Au and *w*Ri are most similar to *w*Mel. Whereas of the phylogenetic supergroup A strains, *w*Ha is the most divergent to *w*Mel, and *w*No belongs to supergroup B [18],[20]. This may suggest that there is a *Wolbachia* feature involved in antiviral protection, which is conserved among strains more closely related to *w*Mel.

With the exception of the Me29 flies infected by *w*Mel, natural host-*Wolbachia* combinations were used. The *D. simulans Wolbachia* strains are known to be associated with different mitochondrial haplotypes [22] and we did not control for host nuclear genetic background which can have an impact on virus infection [7]. As a consequence it is not possible to rule out that intrinsic variability in susceptibility to virus that is linked to the host background has an influence on the outcome of *Wolbachia*-mediated protection in our experiments. Indeed there is variation in the time to death of *Wolbachia*-free *D. simulans* lines used in this study when challenged with DCV (Figure 2), although interestingly these same *Wolbachia*-free lines showed similar time to death when challenged with FHV (Figure 4). Antiviral protection was observed in both *D. melanogaster* and *D. simulans* when infected with *w*Mel. This indicates that antiviral protection mediated by *Wolbachia* can be transferred between different host species.

Since protection against DCV was not seen in all the fly lines infected with the *Wolbachia* strains, we tested whether there is specificity in protection against different viruses. Infection of *D. melanogaster* by *Wolbachia* protected the flies from all RNA viruses tested [7],[8]. Although each of these viruses was a non-enveloped, positive sense RNA virus, the viruses come from a broad spectrum of virus families. Compared to DCV the most divergent of these viruses is FHV. DCV is a member of the *Dicistroviridae* family and has a single genomic RNA that is not capped but is polyadenylated [9]. The genome is a bicistronic mRNA from which the structural and non-structural polyproteins are translated via internal ribosome entry sites [23]–[25]. DCV RNA replication occurs on membranes derived from the golgi [26]. In contrast, the nodavirus FHV genome comprises two mRNA sense RNAs which are capped but not polyadenylated and a third subgenomic RNA is synthesised during replication [27]. FHV genome replication occurs on mitochondrial membranes [28],[29]. Interestingly, although DCV and FHV have distinct infection cycles the same *Wolbachia* strains protected *D. simulans* lines from both DCV and FHV induced mortality. This suggests that the mechanism of protection from virus-induced mortality may be common across diverse viruses, although it is not currently known what the mechanism of viral pathogenesis is in flies infected with either DCV or FHV. It remains to be seen whether the same host-*Wolbachia* combinations that do or do not protect against DCV and FHV have similar outcomes for other viruses, or indeed other types of pathogens.

Concurrent with protection from virus induced mortality in *D. melanogaster* was a delay in accumulation of DCV [8]. Here a similar result was seen with *w*Mel protection in *D. simulans*, the amount of infectious virus accumulated 2 dpi was significantly lower in *Wolbachia* infected flies. By 10 dpi the DCV titre in *Wolbachia* infected flies was similar to the day 2 titre for *Wolbachia*-free flies. It would be tempting to speculate that the resistance to DCV accumulation protects the flies from DCV induced mortality, however, the results observed with the *D. simulans Wolbachia* strains complicate this interpretation. The CO flies infected with *w*Au survived DCV infection beyond 30 dpi, whereas the *Wolbachia*-free flies were clearly susceptible to DCV-induced mortality. *w*Au infected flies had by 10 dpi accumulated high titres of DCV and the virus titre remained high at 30 dpi. This shows that *w*Au infected flies were tolerant of DCV infection, that is the virus accumulated but did not cause mortality [30]. Interestingly, although *w*Ri-infected DSR flies were protected from DCV induced mortality, at 2 dpi there was no difference in virus accumulation in flies with and without *w*Ri. We cannot rule out that accumulation was delayed in *w*Ri-infected flies earlier than 2 dpi.

Taken together our results indicate that *Wolbachia*-mediated antiviral protection could arise in flies in two ways. *Wolbachia* can interfere with the virus infection cycle to delay virus accumulation, that is, it can induce resistance to virus infection in the host. In addition *Wolbachia* infection can protect flies from the pathogenesis associated with virus infection, that is, it can increase host tolerance to virus infection. The processes or mechanisms involved in resistance and tolerance may be the same, independent or overlap. Our results show that *Wolbachia* strains can induce both resistance and tolerance to DCV infection, but importantly prolonged resistance is not a requirement for protection against DCV-induced mortality. These results are consistent with those reported for FHV in *Wolbachia* infected *D. melanogaster*, where there was no difference in FHV accumulation 6 dpi but *Wolbachia* infection protected flies from FHV induced mortality [7].

The strains of *Wolbachia* that mediate antiviral protection were anticipated to be present at higher density in infected flies [31],[32]. We confirmed the density of *Wolbachia* in the particular fly lines used in this study correlated with protection. The density of *Wolbachia* was assayed in whole flies as previous assays have shown that in addition to reproductive tissues somatic tissues are commonly infected with *Wolbachia* [33],[34]. Further experiments controlling the density of a single strain are required to determine if high *Wolbachia* density is a pre-requisite for antiviral protection.

The mechanisms or processes by which *Wolbachia* protects the host from virus are not yet understood. The correlation of high bacterial density of the strains that protect the host suggests that *Wolbachia* density may be important for antiviral protection. Potentially protection may require a threshold of *Wolbachia* density to be exceeded, which would be consistent with protection being a consequence of competition between the two intracellular microbes for limited host resources. Antiviral protection may also be dependent on the distribution of *Wolbachia* between tissue or cell types. *Wolbachia* have been identified in a range of somatic and reproductive tissues in insects and are known to display variable tissue tropism depending on infecting strain and host combination [33]–[35]. Late in infection DCV is widely distributed in *Drosophila* tissues including both reproductive and somatic tissues [36]–[38], giving abundant opportunity for overlap with *Wolbachia* distribution. However, little is known about the spread of virus from the initial infection site or if replication of the virus is equivalent in all of the susceptible tissues. It is possible that there are tissues or cell types that are critical to virus replication or pathogenesis and that *Wolbachia*-mediated protection occurs by exclusion or regulation of virus in these tissues. In addition, if particular tissues are critical for pathogenesis, tolerance may be a result of protection of those tissues.

The relatively close phylogenetic relationships of the strains that do confer antiviral protection compared to non-protective strains, suggests that other features of the *Wolbachia* strains could determine the outcome of virus infection. Protection via both resistance and tolerance could be induced by modulation of host antiviral responses by *Wolbachia*. For example, proteins from the ankyrin family, which can play a role in innate immune pathways, vary considerably both in number and sequence between *Wolbachia* strains [39]–[42]. Interestingly defence against bacterial infection in flies via the melanisation response has been shown to involve both resistance and tolerance effects [43].

*Wolbachia* are able to rapidly invade host populations and are often maintained at high prevalence in these populations [44]. In many cases this is achieved at least in part by *Wolbachia* manipulation of host reproductive systems to increase the prevalence of infected individuals in the host population. For example the *Wolbachia* strains *w*Ri, *w*Ha and *w*No used in this study induce cytoplasmic incompatibility in *D. simulans,* however *w*Au does not manipulate host reproductive systems [45]–[48]. In the absence of strong reproductive parasitism, theory predicts that to be maintained in a host population *Wolbachia* must provide a fitness advantage to the female host (reviewed in [49],[50]). *Wolbachia*-mediated protection from viruses and other pathogens [51] may confer this fitness advantage. It is therefore likely that the interactions between *Wolbachia* and viruses such as DCV impact on the distribution of both microbes in insect populations.

## Materials and Methods

### Viruses

Plaque purified DCV isolate EB [11] and FHV [52] were propagated and purified from DL2 cells [53]. DL2 cells were maintained in Schneider's media supplemented with 10% FBS, 1 x glutamine and 1 x penstrep (Invitrogen) at 27.5°C. Cells grown in 75 cm^2^ flasks were infected with either DCV or FHV at a low multiplicity of infection (<1) and harvested at 4–5 dpi. Cells were lysed by two rounds of freeze-thawing and cell debris removed by centrifugation at 5,000 rpm for 5 min. The virus was purified from the supernatant by pelleting through a 6 ml 10% sucrose cushion at 27,000 rpm at 12°C for 3 hours in a SW28 swing bucket rotor (Beckman). The resuspended virus was layered onto a continuous 10–40% w/v sucrose gradient and centrifuged at 27,000 rpm at 12°C for 3 hours in a SW41 swing bucket rotor (Beckman). The virus-containing fractions were harvested, diluted in 50 mM Tris pH 7.4 and virus was pelleted by centrifugation at 27,000 rpm, 12°C for 3 hours. The virus was resuspended in 50 mM Tris pH 7.4 at 4°C overnight, aliquoted and stored at −20°C. The concentration of tissue culture infectious units (IU) of each virus preparation was determined by replicate TCID_50_ analysis on two separate frozen aliquots, as previously described [8].

### Flies and *Wolbachia*

All *Wolbachia* infected fly lines were obtained from the culture collection in the O'Neill lab and were maintained on standard cornmeal diet at a constant temperature of 25°C with a 12-hour light/dark cycle. The *D. simulans* fly line Me29 is infected with *w*Mel. The *w*Mel infection was established by injection of *Wolbachia* containing cytoplasm from *D. melanogaster* Wien 5 embryos into *D. simulans* NHaTC embryos [21]. The other *D. simulans* lines are naturally infected with *Wolbachia* strains as previously described and are listed in Table 1 [45]–[47],[54].

### Preparation of *Wolbachia-* and virus-free fly lines

Virus-free populations of each of the *Wolbachia* containing fly line were prepared essentially as previously described [55]. Briefly, flies were aged for at least 20 days, transferred to fresh media (supplemented with dry yeast) and allowed to lay eggs for up to 16 hours. The eggs were collected from the surface of the media and treated for 4 minutes in 1.7% (w/v) sodium hypochlorite solution to remove the chorion. After treatment the eggs were thoroughly rinsed with water, transferred to moist filter paper and placed on fresh virus-free media. Virus-free flies were maintained separately from untreated stocks.

To generate fly lines free of *Wolbachia* each virus-free *Wolbachia* infected fly line was treated with 0.03% tetracycline [45]. Following the tetracycline treatment flies were held for more than four generations to recover before being used for experiments.

### Survival bioassays

*Drosophila* were infected with DCV, FHV or mock infected by microinjection of virus or PBS into the upper lateral part of the abdomen. Samples were injected using needles pulled from borosilicate glass capillaries and a pulse pressure micro-injector into 4–7 day old male flies that were anaesthetised with carbon dioxide. For each fly line assayed, three groups of 15 flies were injected with virus and one group of 15 flies were injected with PBS. After injection flies were maintained in vials at a constant temperature of 25°C with a 12 h light/dark cycle and mortality was recorded daily. Mortality that occurred within one day of injection was deemed to be due to injury. Each experiment was replicated using independent cohorts of flies. Survival curves were compared using Kaplan-Meier analysis and log-rank statistics reported (GraphPad Prism). For each assay described in this paper a fresh aliquot of either DCV or FHV was defrosted and diluted to 1×10^8^ IU/ml before use.

### Virus accumulation assays

The accumulation of infectious DCV particles in both *Wolbachia* infected and uninfected flies was measured. For each of the five fly lines, groups of flies with and without *Wolbachia* were injected with DCV as for survival bioassays. At designated times post injection, two pools of four live DCV injected flies were collected and frozen at −20°C. Flies from all *Wolbachia* infected and uninfected fly lines were collected at 2 dpi. For Me29, DSR and CO flies infected with *Wolbachia* samples were also collected at 10 days post injection; for N7NO and DSH containing *Wolbachia* and all tet-treated lines there were not enough live flies remaining at 10 days for collection. For CO-*Wolbachia* flies an additional collection was included at 30 dpi.

Each pool of four flies was homogenised in 100 µl of PBS with two 3 mm beads (Sigma-Aldrich) using a Mini BeadBeater-96 (Biospec Products) for 60 seconds. The homogenates were clarified by centrifuging at 14 K for 8 minutes. The virus–containing supernatant was aliquoted and stored at −20°C. Virus titre was determined using the TCID_50_ assay as previously described [8]. The two replicates for each fly population were assayed on different days to control for between-day variation in TCID_50_ assays. Statistical analysis of the data was done using unpaired t tests to compare the geometric means of the duplicate samples between flies of each line with and without *Wolbachia* at 2 dpi (GraphPad Prism).

### Analysis of *Wolbachia* density

For each fly line 200 eggs were collected and incubated on fresh food with a constant temperature of 25°C for 10 days. Freshly emerged flies were collected for 8 hours, aged to 4 days old and then five male flies from a single collection were pooled. For each fly line a total of 10 pools of flies were collected from independent bottles and the DNA extracted using a DNeasy Blood and Tissue Kit as per manufacturers instructions (Qiagen). The relative ratio of *Wolbachia* to fly genomic DNA was determined by quantitative PCR. Each 10 µl qPCR reaction included 5 µL of Sybr Green qPCR Supermix-UDG (Invitrogen), 1 µL of DNA template and 1 µM each of the forward and reverse primers. Primers for *Wolbachia* were designed from an alignment of the sequence of the WSP genes from all five *Wolbachia* strains (wspFQALL 5′ GCATTTGGTTAYAAAATGGACGA 3′ and wspRQALL 5′ GGAGTGATAGGCATATCTTCAAT 3′) and for the host gene RPS17 (Dmel.rps17F 5′CACTCCCAGGTGCGTGGTAT 3′ and Dmel.rps17R 5′GGAGACGGCCGGGACGTAGT 3′). Reactions were done in duplicate in a Rotor-gene thermal cycler (Corbett Life Sciences) with the following conditions: one cycle of 50°C 2 min, 95°C 2 min, followed by 40 cycles of 95°C 5 sec, 60°C 5 sec, 72°C 10 sec. A third technical replicate was done where necessary and DNA extracted from flies without *Wolbachia* was used as a negative control. Ratios were calculated in Qgene and statistical analysis included Mann-Whitney t test to compare differences of the means.

### Accession numbers

EF423761 wsp *w*Ri; DQ235409 wsp *w*Au; AF020074 wsp *w*No; AF020073 wsp *w*Ha; NM_079278 RPS17.
